# Half of Pulmonary Tuberculosis Cases Were Left Undiagnosed in Prisons of the Tigray Region of Ethiopia: Implications for Tuberculosis Control

**DOI:** 10.1371/journal.pone.0149453

**Published:** 2016-02-25

**Authors:** Kelemework Adane, Mark Spigt, Semaw Ferede, Tsehaye Asmelash, Markos Abebe, Geert-Jan Dinant

**Affiliations:** 1 Department of Medical Microbiology and Immunology, College of Health Sciences, Mekelle University, Mekelle, Ethiopia; 2 Maastricht University/CAPHRI School for Public Health and Primary Care, Department of Family Medicine, Maastricht, the Netherlands; 3 Department of Public Health, College of Health Sciences, Mekelle University, Mekelle, Ethiopia; 4 Armauer Hansen Research Institute, Addis Ababa, Ethiopia; University of Maryland School of Medicine, UNITED STATES

## Abstract

**Introduction:**

Prison settings have been often identified as important but neglected reservoirs for TB. This study was designed to determine the prevalence of undiagnosed pulmonary TB and assess the potential risk factors for such TB cases in prisons of the Tigray region.

**Method:**

A cross-sectional study was conducted between August 2013 and February 2014 in nine prisons. A standardized symptom-based questionnaire was initially used to identify presumptive TB cases. From each, three consecutive sputum samples were collected for acid-fast bacilli (AFB) microscopy and culture. Blood samples were collected from consented participants for HIV testing.

**Result:**

Out of 809 presumptive TB cases with culture result, 4.0% (95% CI: 2.65–5.35) were confirmed to have undiagnosed TB. The overall estimated point prevalence of undiagnosed TB was found to be 505/100,000 prisoners (95% CI: 360–640). Together with the 27 patients who were already on treatment, the overall estimated point prevalence of TB would be 793/100,000 prisoners (95% CI: 610–970), about four times higher than in the general population. The ratio of active to passive case detection was 1.18:1. The prevalence of HIV was 4.4% (36/809) among presumptive TB cases and 6.3% (2/32) among undiagnosed TB cases. In a multivariate logistic regression analysis, chewing Khat (adjusted OR = 2.81; 95% CI: 1.02–7.75) and having had a close contact with a TB patient (adjusted OR = 2.18; 95% CI: 1.05–4.51) were found to be predictors of undiagnosed TB among presumptive TB cases.

**Conclusions:**

This study revealed that at least half of symptomatic pulmonary TB cases in Northern Ethiopian prisons remain undiagnosed and hence untreated. The prevalence of undiagnosed TB in the study prisons was more than two folds higher than in the general population of Tigray. This may indicate the need for more investment and commitment to improving TB case detection in the study prisons.

## Introduction

Finding and successfully treating all tuberculosis (TB) patients is the cornerstone of the Global Strategy to Stop TB [1]. However, a World Health Organization(WHO) report from 2013 indicated that a key obstacle to achieving this goal has been that many people with TB are currently being ‘missed’ by health systems [2]. It is anticipated that the majority of these cases could be among the poorest people of the world [2]. Exploring undiagnosed TB was therefore greatly emphasized by WHO and mentioned as a priority area of research [2].

Prisoners are at a disproportionately high risk of TB and HIV infection [3]. Many people in prisons could have undiagnosed TB, especially in the sub-Saharan African prisons, where the prison cells are extremely overcrowded, health services are very poor, and HIV infection and malnutrition are very prevalent [4,5]. Previous studies in the sub-Saharan African prisons reported TB prevalence rates ranging from 3.5%-5.8% [6–10] with a high TB/HIV co-infection rate. About 44% of prisoners with TB in a South African prison [9] and 37% in Zambian prisons [10] were co-infected with HIV.

In Ethiopia, prisoners are incarcerated in an overcrowded and poorly ventilated environment [11]. The prison health services are poorly organized, lacking skilled manpower and laboratory facilities for TB diagnosis [11]. There is no protocol for screening prisoners on admission, during incarceration or on discharge. TB diagnosis relies merely on a referral of prisoners to health facilities outside prisons which usually imposes a serious inconvenience to patients [11]. As a consequence, a number of prisoners with TB in Ethiopian prisons could remain undiagnosed which is a serious risk not only for other prisoners but, through visitors and the prison personnel, also has implications for the general population [12]. Estimating the magnitude of undiagnosed TB and identifying factors contributing for acquiring TB can help assessing the level TB control program in a given setting and is crucial in designing strategies that will improve the TB control programs. Despite this, only limited work has been done on TB or TB/HIV in Ethiopian prisons. Previous studies from Gondar [13] and Eastern Ethiopian prisons [14] reported a seven and nine-fold higher proportion of TB than in the general population, respectively. However, these studies were limited in scope in that the former was focused only on smear-positive cases while the latter didn't include HIV investigation. Besides, in Ethiopia, the socioeconomic, lifestyle and environmental conditions differ across regions [15] and could affect the distribution of TB diseases. The burden of TB in prison of the Tigray region is not yet defined. Therefore, this study aimed at determining the prevalence of undiagnosed pulmonary TB (henceforth called undiagnosed TB) and assessing the potential risk factors for such TB cases in nine prisons of the Tigray region.

## Methods and Materials

### Study setting

This study was conducted in prisons of the Tigray Regional State, Northern Ethiopia. Ethiopia is one of the countries with a high TB/HIV burden in the sub-Saharan Africa [2]. Ethiopia had an officially registered prison population of 112,361 (136/100,000 persons) in 2010 [16], which is higher than the imprisonment rates observed in some sub-Saharan African countries such as in Kenya (121/100,000 persons), and Malawi (76/100,000 persons). But, this is lower than the imprisonment rates in South Africa (294/100,000 persons), and Rwanda (492/100,000 persons) [16]. There were a total of nine prisons in the Tigray region located in the cities: Adigrat, Adwa, Alamata, Axum, Humera, Maychew, Mekelle, Shire and Wukro. All the nine prisons were included in the study. The study was focused only in prisons of the Tigray region of Ethiopia merely due funding limitations.

### Study design and population

This was a cross-sectional study conducted in nine prisons of the Tigray Regional State between August 2013 and February 2014. Prisoners were our study populations; prison guards were not included due to resource limitations. Inclusion criteria for initial screening were: providing written informed consent, being an age of ≥ 18 years and not being treated for TB at the time of the screening. Participants providing written consent for HIV testing and having had clinical symptoms of pulmonary TB (cough of ≥ 2 weeks and sputum production with at least one of the other symptoms such as night sweating for at least 2 weeks, fever for at least two weeks, chest pain for at least 2 weeks and /or unintentional weight loss) provided sputum samples for subsequent bacteriological testing.

### Sampling procedures and specimen collection

At the time of the study, there were about 9326 prisoners held in nine prisons of which 27 were already on anti-TB treatment. All prisoners who were not taking anti-TB treatment (n = 9299) were initially screened for a cough of any duration by prison nurses through a cell to cell visit in each prison. Then, senior nurses, recruited from TB clinics of nearby hospitals, performed further screening using a symptom-based questionnaire adapted from the international guideline of TB screening protocol in prisons [17]. A presumptive TB case was defined as a participant with symptoms of TB including having a cough of ≥ 2 weeks and able to produce sputum with at least one of the other symptoms such as night sweating for at least 2 weeks, fever for at least two weeks, chest pain for at least 2 weeks and /or unintentional weight loss. Prisoners with a cough of < 2 weeks, and those with ≥ 2 weeks but unable to produce sputum were not considered as presumptive TB cases and hence were excluded. Accordingly, about 1231 presumptive TB cases were identified. However, the number of presumptive TB cases included for subsequent bacteriological testing was 844 (68%). A proportional stratified sampling technique was employed to assign the required number of samples from each prison according to their total number of prisoners. The number of participants included ranged from 49 in Adawa to 231 in Mekelle prison and they were enrolled following a simple random sampling technique. The remaining 387 presumptive TB cases were not included for bacteriological testing due to financial constraints to undertake culture. However, the final estimation was calculated by considering these untested presumptive TB cases by extrapolation in which the extrapolation was done by assuming a same prevalence of TB among 387 untested presumptive TB cases as the tested ones.

Data were collected on the socio-demographic characteristics and health status of prisoners including age, sex, educational status, cigarette smoking and Khat chewing habit, duration of imprisonment, time of symptom development, and history of sharing a cell with previously diagnosed TB patients (close contact history) using a structured questionnaire. A close contact was defined in this study as a person with prolonged frequent contact exposure (at least a total of 8 hours of direct exposure during the period of infectiousness preceding the diagnosis of the index case) [18]. Information on the referral system was also collected by interviewing presumptive TB cases that had a history of reporting TB-like complaints to the prison staff for a referral. A checklist was used to collected data on the conditions of prisons. To minimize recall bias for some questions such as time of symptom development, we used a carefully constructed questionnaire with specific details and the participants were given enough time before answering the questions. The body weight and height was also measured. Body mass index (BMI) was calculated for each participant and their nutritional status was categorized according to the WHO standards [19]. Then, three consecutive early morning sputum samples were collected from each participant and samples were stored at -20°C for a maximum of 2–3 weeks until transported in an ice box to the Armauer Hansen Research Institute (AHRI) in Addis Ababa for culturing. We collected three early morning sputum samples instead of the routine spot-morning-spot algorithm in order to maximize the detection of undiagnosed TB cases because of that early morning sample has been shown to have a high incremental yield than spot samples [20].

### Laboratory methods and diagnostic criteria

Direct microscopic examination of the sputum samples for AFB and culturing was performed at AHRI using solid media. Prior to culturing, sputum samples were decontaminated by the Petroff's method following the standard operational procedures. In brief, an equal volume of sputum sample was mixed with an equal volume of 4% NaOH (Sodium hydroxide) and concentrated at 3000 rpm for 15 minutes. The sediment was neutralized with 2N HCL (Hydrochoric acid), in the presence of phosphate buffer, using phenol red as an indicator to make the inoculums for culture. Then, 3 Lowenstein-Jensen slants, two containing 0.75% glycerol and one containing 0.6% pyruvate were inoculated per each sputum sample. Culture tubes were incubated at 37°C and growth was checked weekly for up to 8 weeks. A bacteriologically confirmed TB case was defined as a presumptive TB case with two smear-positive sputum samples by the direct microscopy and/or positive culture for *M*.*tuberculosis*. Undiagnosed TB was defined in this study as a bacteriologically confirmed TB in a person not being treated at enrollment. Confirmed TB patients were referred to a nearby health facility and were treated as per the national guidelines.

Prior to HIV testing, all consenting prisoners were offered a pre-test HIV counseling by the screening nurses. Testing was conducted using the rapid HIV test kits according to the current National algorithm recommended by the Federal Ministry of Health of Ethiopia. Samples were tested first with HIV (1+2) Antibody Colloidal Gold (KHB). Positive samples were confirmed with Stat-Pak while discordant results were resolved by HIV-1/2 Unigold Recombinant assay. Post-test HIV counseling was also done and the result was given only for those wanting to know their HIV status with linkage to health facilities for appropriate care.

### Data analysis

Data were entered using Epi Data entry version 3.1 software and analyzed using SPSS version 21. Our outcome variable of interest was comparing prisoners with undiagnosed TB with those without TB in the subset of presumptive TB cases. The analysis of risk factors was limited to the undiagnosed TB cases; all the 27 TB patients that were already on treatment were not included. Bivariate and multivariate logistic regression analysis was performed to examine the association of independent variables with acquiring undiagnosed TB. Covariates with p-values of ≤ 0.25 in a bivariate analysis and collinearity matrix index of ≤ 0.7 were considered for inclusion in the multivariate model. However, due to the small number of outcomes (n = 32), only those variables strongly related with bacteriologically confirmed TB (P < 0.1) in the bivariate analysis were included in the final model. P values of ≤ 0.05 were considered as statistically significant.

### Ethical issues

The study was approved by ethical review committees of the College of Health Sciences, Mekelle University, and AHRI/ALERT (Armauer Hansen Research Institute/All African Leprosy) ethics review committees. All participants were enrolled after they provided us a written informed consent including for HIV testing. Presumptive TB cases that were not included in the study due to financial constraints were referred for regular health care to nearby hospitals. For all illiterate participants, data collectors informed each respondent and confirmed the willingness of the participants by signing on the informed consent sheet. The consent procedure for these illiterate participants was also approved by both ethics review committees. A brief education was provided for those prisoners that refused to know their HIV status so as to improve their awareness.

## Results

### Conditions of prisons

During our study, we observed that TB control was inadequate in the study prisons. All the nine prisons had a clinic, but only two clinics (of Mekelle and Adigart prisons) have a building with more than one room. All clinics were staffed by nurses, but there were no physicians. None of the clinics had services for sputum smear microscopy and there was no protocol for entry, periodic or exist screening of prisoners for TB. TB diagnosis was performed following the passive case finding strategy which relied on a referral of prisoners to health facilities outside prisons. However, we also observed the lack of an adequate referral system, meaning that prisoners were only referred after repeated complaints of TB symptoms, or when they developed severe signs and symptoms. Materially, the inmates were poorly housed; inmates slept on mats or on a very poor quality mattress on the floor. The number of prisoners per room varied for each prison, ranging from 18–172 with an average of 64. Rooms were generally overcrowded and had poor ventilation systems. Most rooms had a narrow sized window while some others had no windows at all.

### Prevalence of undiagnosed pulmonary TB and HIV infection

**Fig 1:**
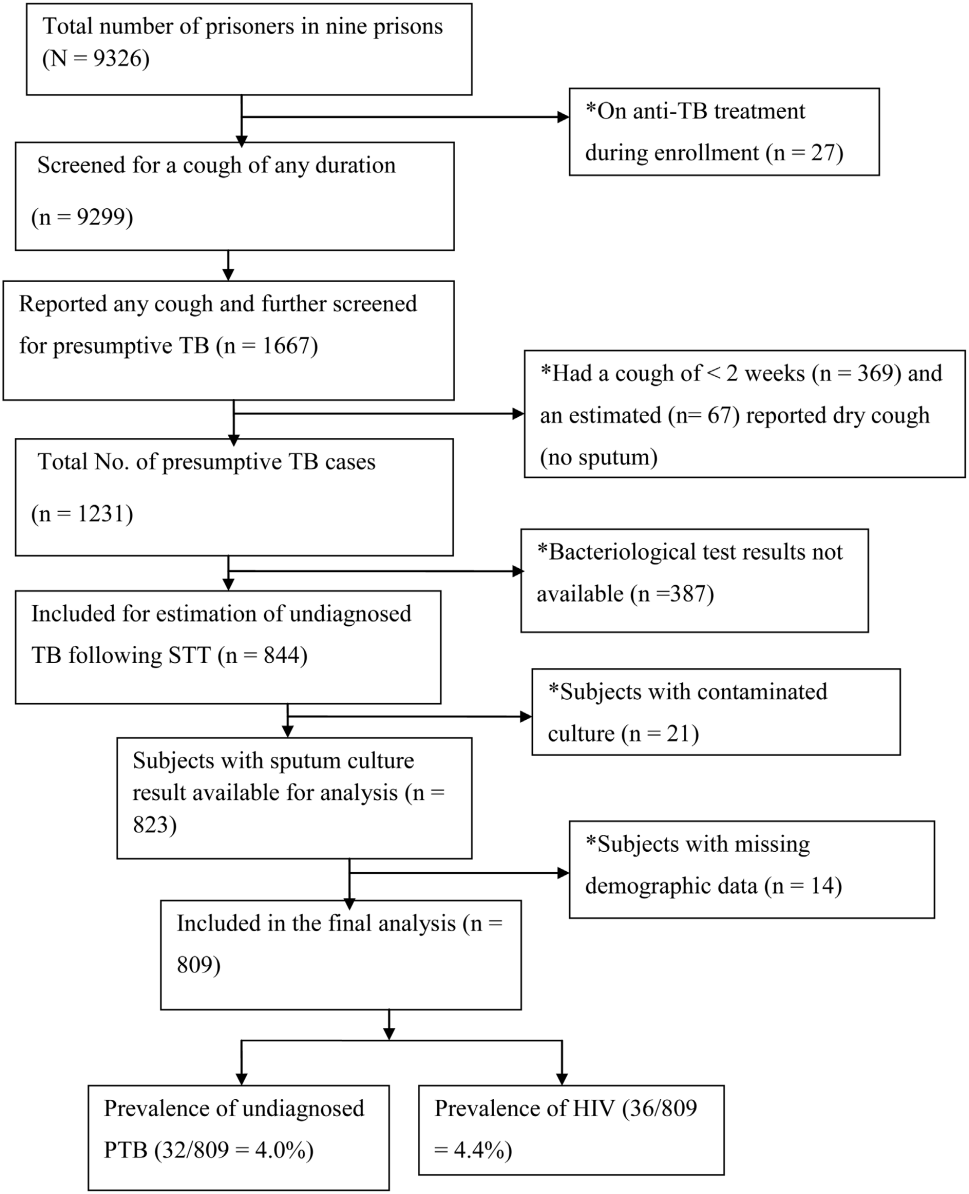
Flow chart of study inclusion, exclusion and diseases (PTB and HIV) outcomes. * = Excluded; SST = Stratified sampling technique; PTB = Pulmonary TB; HIV = human immunodeficiency virus.

Fig 1 summarizes the study recruitment flow and undiagnosed TB outcomes. Of the 844 presumptive TB cases enrolled, complete data with interpretable culture results were available for 809 (96%) cases in which 4.0% ([32/809]; 95% CI: 2.65–5.35) were confirmed to have undiagnosed TB. Assuming that the prevalence among the 387 untested prisoners with a cough was also 4%, the estimated point prevalence of undiagnosed TB in the total prison population would be 32+15/9299 = 0.505% or 505/100,000 prisoners (95% CI: 360–640). Together with the 27 patients who were already on treatment, the estimated point prevalence of TB would be 32 +15 +27/9326 = 0.793% or 793/100,000 prisoners (95% CI: 610–970), about four times higher than in the general population. The ratio of newly detected TB cases through active case finding to those detected following passive case detection (to those already on treatment) was about 1.18:1 (32/27) indicating that for every TB case that is found, at least one case is undetected. Of the 32 undiagnosed TB cases, the majority (84.4% [27/32]) were smear-negative /culture positive cases while 5 cases were smear-positive /culture positive. There were no smear-positive /culture negative cases. Twenty-seven were newly infected cases and 5 of the undiagnosed TB cases reported a previous TB treatment. All cases were male prisoners; all 21 female prisoners enrolled were culture negative. The majority (75% [24/32]) of undiagnosed TB cases responded that they had a history of reporting their TB-like symptoms to the prison health personnel for one or more time. The prevalence of HIV was 4.4% (36/809) among presumptive TB cases and 6.3% (2/32) among undiagnosed TB cases. Both of these HIV positive cases with undiagnosed TB were smear-negative.

### Characteristics of presumptive TB cases and relation to undiagnosed pulmonary TB

Of 809 participants, the majority (788 [97%]) were men. The mean age of the presumptive TB cases was 33 years (range 18–82 years). The average duration of imprisonment at the time of the screening was 2 and half years (range 1 month-21 years), the majority (63%) of undiagnosed TB cases were imprisoned for more than a year. More than three-forth (82%) of the presumptive TB cases developed a cough after entering the prison and the majority (516 [64%]) had a cough for 4 weeks or longer. The median BMI among the presumptive TB cases was 19.9 kg/m^2^ (range 13.7–31.8) and 203 (25%) of them were malnourished (BMI < 18.5 kg/m^2^). About 76 (9.4%) of the presumptive TB cases had chewed Khat and 171 (21.2%) smoked cigarettes.

The prevalence of undiagnosed TB in the study population (presumptive TB cases) did not significantly differ across the nine prisons included in this study (Table 1).

**Table 1:** The distribution of undiagnosed TB among presumptive TB cases by prison sites in Northern Ethiopia as analyzed by the binary logistic regression (n = 809).

Prison site	Presumptive TB cases included in the analysis, n (%) (N = 809)	Confirmed undiagnosed TB cases, n (%) (N = 32)	OR (95% CI)	P-value
**Axum**	**59 (7.3)**	**1 (1.7)**	**Ref.**	
**Mychew**	**66 (8.2)**	**0 (0)**	**0.00**	**0.98**
**Adwa**	**44 (5.4)**	**1 (2.3)**	**1.3 (0.08–22.18)**	**0.83**
**Adigrat**	**97 (11.9)**	**3 (3.1)**	**1.9 (0.18–18.22)**	**0.59**
**Shire**	**131 (16.2)**	**6 (4.6)**	**2.9 (0.33–23.65)**	**0.35**
**Humera**	**68 (8.4)**	**3 (4.4)**	**2.7 (0.27–26.45)**	**0.40**
**Alamata**	**58 (7.2)**	**2 (3.4)**	**2.1 (0.18–23.49)**	**0.56**
**Mekelle**	**231 (28.6)**	**13 (5.6)**	**3.5 (0.44–26.99)**	**0.24**
**Wukro**	**55 (6.8)**	**3 (5.5)**	**3.3 (0.34–33.17)**	**0.30**

CI = Confidence interval; OR = Odds ratio; Ref. = Reference

The relationships of demographic and clinical risk factors with undiagnosed TB among the presumptive TB cases are presented in Tables 2 and 3, respectively. There was a significant association between chewing Khat and acquiring undiagnosed TB; the risk of acquiring undiagnosed TB among chewers being about 3 times higher than non-chewers (OR = 3.47; 95% CI: (1.51–8.03); P = 0.004). Having a history of close contact with TB patients was also associated with undiagnosed TB (OR = 2.32; 95% CI: 1.13–4.76; P = 0.02). On the other hand, the potential socio-demographic risk factors including cigarette smoking, history of sharing a cell with TB patients, age variation and duration of incarceration were not significantly associated with undiagnosed TB. Similarly, there was no association between undiagnosed TB and the potential clinical risk factors such as duration of a cough, a previous history of reporting TB-like complaints to the prison staff, BMI, and HIV infection status.

**Table 2:** Bivariate analysis of the socio-demographic and prison related factors for undiagnosed TB outcome among presumptive TB cases in Northern Ethiopian prisons (n = 809).

Variable	Presumptive TB cases included in the analysis, n (%)	Confirmed undiagnosed TB cases, n (%)	Presumptive TB cases without undiagnosed TB, n (%)	OR (95% CI)	P-value
**Age (years)**					
**18–44**	**655 (81.0)**	**28 (4.3)**	**627 (95.7)**	**1.68 (0.58–4.85)**	**0.34**
**≥45**	**154 (19.0)**	**4 (2.6)**	**150 (97.4)**	**Ref.**	
**Educational status**					
**No formal education**	**209 (25.8)**	**5 (2.4)**	**204 (97.6)**	**Ref.**	
**Have formal education**	**600 (74.2)**	**27 (4.5)**	**573 (95.5)**	**1.92 (0.73–5.05)**	**0.18**
**Cigarette smoking (current)**					
**No**	**638 (78.8)**	**21 (3.3)**	**617 (96.7)**	**Ref.**	
**Yes**	**171 (21.2)**	**11 (6.4)**	**160 (93.6)**	**2.02 (0.95–4.28)**	**0.06**
Chewing Khat* **(current)**					
**No**	**733 (90.7)**	**24 (3.4)**	**709 (96.6)**	**Ref.**	
**Yes**	**76 (9.)**	**8 (9.3)**	**68 (90.7)**	**3.47 (1.51–8.03)**	**0.004**
**Sharing a cell with a TB patient**					
**No**	**620 (76.6)**	**23 (3.7)**	**597 (96.3)**	**Ref.**	
**Yes**	**189 (23.4)**	**9 (4.8)**	**180 (95.2)**	**1.29 (0.59–2.86)**	**0.52**
**TB contact history**					
**No**	**600 (74.2)**	**18 (3.0)**	**582 (97.0)**	**Ref.**	
**Yes**	**209 (25.8)**	**14 (6.7)**	**195 (93.3)**	**2.32 (1.13–4.76)**	**0.02**
**Duration of incarceration**					
**<12 months**	**324 (40.0)**	**12 (3.7)**	**312 (96.3)**	**Ref.**	
**≥12 months**	**485 (60.0)**	**20 (4.1)**	**465 (95.9)**	**1.12 (0.54-.232)**	**0.76**
**No of prisoners per cell**					
**≤50**	**261 (32.2)**	**8 (3.0)**	**253 (97.0)**	**Ref.**	
**>50**	**548 (67.7)**	**24 (4.4)**	**524 (95.6)**	**1.45 (0.64–3.26)**	**0.37**

TB = tuberculosis; OR = Odds ratio; CI = Confidence interval;

* = *Catha edulis*;

Ref. = Reference

**Table 3:** The association of clinical characteristics of presumptive TB cases with undiagnosed TB outcome in Northern Ethiopian prisons by bivariate analysis in the binary logistic regression (n = 809).

Variable	Presumptive TB cases included in the analysis, n (%) N = 809	Confirmed undiagnosed TB cases, n %) N = 32	Presumptive TB cases without undiagnosed TB, n (%) N = 777	OR (95% CI)	P-value
**Chest pain**					
**No**	**182 (22.5)**	**4 (2.2)**	**178 (97.8)**	**Ref.**	
**Yes**	**627 (77.5)**	**28 (4.5)**	**599 (95.5)**	**2.08 (0.72–0.61)**	**0.17**
**Night sweating**					
**No**	**166 (20.5)**	**4 (2.4)**	**162 (97.6)**	**Ref.**	
**Yes**	**643 (79.5)**	**28 (4.4)**	**615 (95.6)**	**1.85 (0.64–5.33)**	**0.26**
**Duration of cough**					
**<4 weeks**	**293 (36.2)**	**10 (3.4)**	**283 (96.6)**	**Ref.**	
**≥4 weeks**	**516 (63.8)**	**22 (4.3)**	**494 (95.7)**	**1.26 (0.58–2.69)**	**0.55**
**Time of occurrence of cough**					
**Before imprisonment**	**146 (18.1)**	**7 (4.8)**	**139 (95.2)**	**1.29 (0.55–3.03)**	**0.57**
**After imprisonment**	**663 (81.9)**	**25 (3.8)**	**638 (97.2)**	**Ref.**	
**Previous TB-like symptom report** *					
**No**	**282 (34.9)**	**8 (25.0)**	**274 (97.2)**	**Ref.**	
**Yes**	**527 (65.1)**	**24 (75.0)**	**503 (95.4)**	**1.63 (0.72–3.69)**	**0.24**
**BMI**					
**<18.5**	**203 (25.0)**	**12 (6.0)**	**191 (94.0)**	**1.84 (0.88–3.84)**	**0.11**
**≥18.5**	**606 (75.0)**	**20 (3.3)**	**586 (96.7)**	**Ref.**	
**HIV infection**					
**No**	**773 (95.6)**	**30 (3.9)**	**743 (96.1)**	**Ref.**	
**Yes**	**36 (4.5)**	**2 (5.5)**	**34 (94.5)**	**1.46 (0.33–6.34)**	**0.62**
**Previous history of TB treatment**					
**No**	**681 (84.2)**	**27 (3.9)**	**654 (96.1)**	**Ref.**	
**Yes**	**128 (18.8)**	**5 (3.9)**	**123 (96.1)**	**1.06 (0.56–3.15)**	**0.92**

* = Previous reporting of TB-like symptoms to the prison personnel for one or more time;

TB = Tuberculosis; BMI = Body mass index; OR = Odds ratio; CI = Confidence interval; Ref. = Reference

After fitting into the multivariable logistic regression model, chewing Khat (adjusted OR = 2.81; 95% CI: 1.02–7.75; P = 0.04) and having had close contact with a TB patient (adjusted OR = 2.18; 95% CI: 1.05–4.51; P = 0.03) remained independently associated with undiagnosed TB (Table 4).

**Table 4:** Multivariate logistic regression model showing risk factors associated with undiagnosed TB among the presumptive TB cases Northern Ethiopian prisons (n = 809).

Variable	Presumptive TB cases included in the analysis, n (%)	Confirmed undiagnosed TB cases, n %)	Presumptive TB cases without undiagnosed TB, n (%)	AOR (95% CI)	P-value
**Cigarette smoking**					
**No**	**638 (78.8)**	**21 (3.3)**	**617 (96.7)**	**Ref.**	
**Yes**	**171 (21.2)**	**11 (6.4)**	**160 (93.6)**	**1.25 (0.51–3.12)**	**0.62**
Chewing Khat*					
**No**	**733 (90.7)**	**24 (3.4)**	**709 (96.6)**	**Ref.**	
**Yes**	**76 (9.3)**	**8 (9.3)**	**68 (90.7)**	**2.81 (1.02–7.75)**	**0.04**
**TB contact history**					
**No**	**600 (74.2)**	**18 (3.0)**	**582 (97.0)**	**Ref.**	
**Yes**	**209 (25.8)**	**14 (6.7)**	**195 (93.3)**	**2.18 (1.05–4.51)**	**0.03**

TB = Tuberculosis; AOR = Adjusted odds ratio; CI = Confidence interval;

* = Catha edulis;

Ref. = Reference

## Discussion

This study revealed that at least half of symptomatic pulmonary TB cases in Northern Ethiopian prisons remain undiagnosed and hence untreated, which is an important public health concern. Out of 809 presumptive TB cases with culture result, 4.0% (95% CI: 2.65–5.35) were confirmed to have a bacteriologically positive undiagnosed TB. The overall estimated point prevalence of undiagnosed TB was found to be 505/100,000 prisoners (95% CI: 360–640). This figure is more than two folds higher than the estimated prevalence of undiagnosed TB in the general population of Tigray (216/100,000 population) [21].

Previous surveys from the sub-Saharan African prisons reported TB prevalence rates ranging from 3.5%-5.8% [6–8] (1.9% from Eastern Ethiopian prisons) [14]. Although it might be difficult to directly compare our figure with these findings because of differences in study design (some were longitudinal) [9], sampling strategies, and screening methodologies (some used X-ray and symptom-based screening) [9,10], our figure is much lower than these reports. One of the reasons for this difference could be due to the differences in the burden of TB in the general populations as these countries had a higher prevalence of TB than Ethiopia [2,22]. Our finding (0.5%) is also much lower than that observed in a prison of Gondar in the northwest Ethiopia (1.5%) [13], especially if we consider that the latter was limited only to smear-positive cases. This discrepancy could partly be attributed to the methodological difference between the two studies in which the criteria to consider as presumptive TB case was cough of ≥ one week in the Gondar study whereas, in our study the criteria was cough of ≥ 2 weeks with at least other symptoms such night seating for at least 2 weeks and may affect the sensitivity of the screening. Moreover, the Gondar study included both pulmonary and extra-pulmonary TB cases while our study was limited only to pulmonary TB cases.

According to the WHO 2014 report, Ethiopia has already achieved the Millennium Development Goals (MDGs) of halving TB incidence, prevalence, and mortality by 2015 indicating a progress on TB control [22]. Together with the rising rates of MDR-TB [23], however, the presence of undiagnosed TB in Ethiopian prisons could disrupt this progress as TB could spread to the community through visitors, prison staff and discharged prisoners. The national TB control program should, therefore, emphasize Ethiopian prisons.

In this study, the majority (84% [27/32]) of undiagnosed TB cases were smear-negative. This figure is unexpectedly high, but we trust our findings as we assured the quality of the smear microscopy in that up-to-date operational procedures were used and two skilled laboratory technologists examined each slide preparation independently. Moreover, our finding is in keeping with studies that show a higher proportion of TB cases detected through active case finding are smear-negative cases when compared with those identified in the passive case finding strategy [9,24,25] imposing a diagnostic challenge to health care workers. Smear-negative cases are also responsible for a substantial proportion of TB transmission (13% to 41%) [26,27].

This study identified two important risk factors for undiagnosed TB; chewing Khat and having had close contact with a TB patient. However, we should mention that risk factors for undiagnosed TB could also be risk factors for acquiring TB in general (whether bacteriologically confirmed or not) as we compared newly diagnosed bacteriologically confirmed TB with non-TB and not undiagnosed with those already diagnosed. Though having had close contact with a TB patient was associated with undiagnosed TB, sharing a cell with a TB patient was not. This is due to the fact that TB contact history in our study included not only those having had contact while in prison, but also those having had contact history before they entered the prison. In this study, three-fourths of TB cases that chewed Khat developed a cough while in prison. In a previous study in Eastern Ethiopian prisons, chewing Khat was not associated with TB [14]. However, a human rights analysis report indicated that chewing Khat significantly contributed to the rise of TB cases in Somalia [28]. One of the explanations for the association could be that people are more exposed to disease transmission due to the fact that they chew Khat in groups in poorly ventilated rooms for many hours. Given that chewing Khat is becoming a common practice in Ethiopia, it could become a significant risk for TB transmission, particularly in overcrowded settings such as prisons and refugee camps. Hence, providing health education focusing on this problem is important to reduce TB transmission.

The HIV prevalence among undiagnosed TB cases in this study (6.3%) is much lower than a previous report from the prison of Gondar (34.6%) [13] and other studies in the sub-Saharan African such as 44% in a South African prison and 37% in Zambian prisons [10]. The difference with other sub-Saharan African prisons could be explained by variations in the burden of HIV in the general populations in that South Africa and Zambia had 8–10 times higher HIV prevalence than Ethiopia [29,30]. The discrepancy with the prison of Gondar might partly be due to differences in the number study sites and sample size in that we included nine prisons while this study was conducted in one prison facility in the city of Gondar which could be in a high burden area. The association between TB and HIV infection is a well-documented phenomenon in the general population [31]. Some studies have also shown this association in the prison population. A study from Cameron prison showed an association between prevalent TB (including that on treatment) but not with undiagnosed TB while studies from Gondar [13] and South African prisons demonstrated an association between undiagnosed TB and HIV infection [9]. In our study, HIV infection was not associated with the presence of undiagnosed TB. The relatively short duration of our study (7 months) might have limited the ability to show an association with HIV infection. Compared to the general population, HIV infection may be a less important risk factor for acquiring TB where the risk among HIV-negative prisoners is also high.

Although the association between time of occurrence of a cough and undiagnosed TB was not statistically significant, the majority (78% [25/32]) of undiagnosed TB cases developed a cough after entering the prison. About 69% (22/32) had coughed for ≥ 4 weeks; some prisoners even have been coughing for more than 2 years. This extended lag time could be due to the weak referral system in the study prisons. We found out that prisoners in our study sites were referred only after repeated complaints of TB symptoms, or when they developed severe signs and symptoms. This would contribute to the ongoing spread of TB within the prison and to the community. However, there was no statistically significant association between duration of imprisonment and undiagnosed TB due to the fact that a substantial proportion of patients in our study (37.5% [12/32]) acquired TB in a shorter period after imprisonment (during the first 11 months of their imprisonment). The overcrowded and poorly ventilated cells, lengthy imprisonment (62.5% of undiagnosed TB cases were imprisoned for a year or more), the increased susceptibility of prisoners (due to poor nutrition and poor sanitation) and the very poor health care system exacerbate the problem in the study prisons. Hence, if we want to see our TB control efforts succeeded, much more attention should be given to introducing and strengthening TB diagnostic services in Ethiopian prisons. To a minimum, the study prisons will need to introduce sputum microscopy service, undertake entry screening, at least, using TB symptom questionnaire, promote a symptom-based screening of prisoners, strengthen the referral system for all prisoners with TB symptoms and ensure segregation and treatment of TB patients.

However, studies have shown that a considerable proportion of TB patients without symptoms were diagnosed with pulmonary TB indicating the insufficiency of symptom-based screening to capture undiagnosed TB cases [32,33]. For effective TB control in prisons, international guidelines recommend X-ray screening in addition to standardized symptom screening for all prisoners entering a high TB risk prisons [17]. A systematic review also indicated that the application of X-ray and symptom questionnaire screening yields better detection of TB cases and suggests the need to incorporate this algorithm in prisons of low/middle-income countries (LMIC) [34]. However, the feasibility and cost-effectiveness of X-ray screening (with or without symptoms) is still questionable in resource-limited prisons settings. Given the high sensitivity of X-ray screening as compared to symptom-based screening alone [35], the government and/ or non-governmental organizations (NGOs) will need to invest in helping evaluate the feasibility and cost-effectiveness of this algorithm and introduce it as a complementary screening tool in addition to the symptom-based inquiry.

New point-of-care TB tests could also benefit Ethiopian prisons. In this regard, the GeneXpert MTB/RIF has proven to be feasible, accurate and effective, giving a sensitivity 97% and specificity 99.2% in an analysis conducted in the general population of five distinct LMIC sites [36]. However, the feasibility and cost-effectiveness of this technology as a routine point-of-care diagnostic test in prisons of resource-limited settings (low-income countries) is not well established and needs a full investigation.

### Strengths and Limitations

One may argue that taking a random sample from the 9299 prisoners (the total number of prisoners minus those already on TB treatment), would be a better strategy to quantify the prevalence of undiagnosed TB. However, we would be missing those presumptive TB cases that should immediately be detected and treated. We tried to undertake a mass screening strategy considering the importance of not missing cases, and the high prevalence of TB in this population. But, as we found more presumptive TB cases than expected, we faced financial constraints and hence culture was done for about 68% presumptive TB cases. The remaining presumptive TB cases were also not left as they were; they were immediately referred to nearby health facilities for diagnosis and immediate treatment. The fact that we had extrapolated positive culture results for the presumptive TB cases that weren't able to obtain cultures was a limitation of this study. However, as we used a representative sample, we trust that our final estimate is accurate. Moreover, we didn't include chest X-ray examination; instead, we relied on symptomatic screening and hence some prisoners that were unable to produce sputum and those non-symptomatic TB cases which could have been detected by chest X-ray might have been missed. The relatively short study duration and the fact that the duration of a cough had to be at least 2 weeks were also limitations of our study.

## Conclusions

This study revealed that more than half of symptomatic pulmonary TB cases in Northern Ethiopian prisons remain undiagnosed and hence untreated with a more than two-fold higher prevalence of undiagnosed TB than in the general population of Tigray. Chewing Khat and TB contact history were found to be predictors of undiagnosed TB among presumptive TB cases. The squalid prison conditions such as overcrowding, poor ventilation system and absence of segregation could contribute to the ongoing and enhanced transmission of TB within the prison and to the community. This implies that there is an urgent need for more investment and commitment to improving TB case detection in the study prisons.
